# Structural Analysis of Mo Thin Films on Sapphire Substrates for Epitaxial Growth of AlN

**DOI:** 10.3390/mi14050966

**Published:** 2023-04-28

**Authors:** Jihong Kim, Youngil Kim, Sung-Min Hong

**Affiliations:** 1 Department of Electrical Engineering, Yeungnam University, Gyeongsan 38541, Republic of Korea; jihongkim@yu.ac.kr; 2 School of Electrical Engineering, Korea University, Seoul 02841, Republic of Korea; kimy9482@korea.ac.kr; 3 Smart Sensor Research Center, Korea Electronics Technology Institute, Seongnam 13509, Republic of Korea

**Keywords:** molybdenum, aluminum nitride, epitaxial growth

## Abstract

Aluminum nitride (AlN) thin film/molybdenum (Mo) electrode structures are typically required in microelectromechanical system applications. However, the growth of highly crystalline and *c*-axis-oriented AlN thin films on Mo electrodes remains challenging. In this study, we demonstrate the epitaxial growth of AlN thin films on Mo electrode/sapphire (0001) substrates and examine the structural characteristics of Mo thin films to determine the reason contributing to the epitaxial growth of AlN thin films on Mo thin films formed on sapphire. Two differently oriented crystals are obtained from Mo thin films grown on sapphire substrates: (110)- and (111)-oriented crystals. The dominant (111)-oriented crystals are single-domain, and the recessive (110)-oriented crystals comprise three in-plane domains rotated by 120° with respect to each other. The highly ordered Mo thin films formed on sapphire substrates serve as templates for the epitaxial growth by transferring the crystallographic information of the sapphire substrates to the AlN thin films. Consequently, the out-of-plane and in-plane orientation relationships among the AlN thin films, Mo thin films, and sapphire substrates are successfully defined.

## 1. Introduction

Micromachining technology is widely used for the development of micro/nanostructured materials and devices. Microelectromechanical systems (MEMS) based on micromachining technology are promising for applications in various industries including electronics, energy, automotive, biomedical, aerospace, and robotics. Many types of materials have been investigated for MEMS applications, and among them, aluminum nitride (AlN) has received considerable attention owing to its versatile and unique properties [1,2,3,4,5,6,7,8,9,10]. AlN is a III–V compound semiconductor with a direct wide bandgap of 6.2 eV. Therefore, AlN has been considered an optoelectronic material for ultraviolet (UV) light-emitting diodes (LED) and UV detectors [11,12]. In addition, AlN is useful in high-temperature and high-power electronics owing to its high-temperature stability, high thermal conductivity, and large dielectric breakdown field [13,14]. The piezoelectric nature of AlN renders it suitable for various MEMS devices such as bulk acoustic wave (BAW) resonators, thin film bulk acoustic resonators (FBAR), surface acoustic wave (SAW) devices, energy harvesters, microphones, and piezoelectric micromachined ultrasonic transducers (pMUT) [15].

In many cases, AlN is used as a thin film for MEMS applications. Therefore, the deposition of AlN thin films on metal layers is necessitated because most electronic devices based on the dielectric and piezoelectric properties of AlN thin films require underlying conducting layers as bottom electrodes. In this regard, the growth of high-quality AlN thin films on several metal layers have been investigated extensively [16,17,18,19,20]. Among various metals, molybdenum (Mo) is typically used as the bottom electrode for AlN thin films due to its advantages, such as low resistivity, low density, high acoustic impedance, insignificant difference in the thermal expansion coefficient, and good adhesion to AlN [21,22,23,24,25]. Despite these advantages, the growth of highly crystalline and *c*-axis-oriented AlN thin films on Mo electrodes remains challenging because of their structural properties. AlN with a wurtzite hexagonal structure preferentially grows along the *c*-axis, which can be enhanced on metal surfaces that exhibit hexagonal symmetry, such as Pt (111), Al (111), and Ti (0001). However, Mo exhibits a body-centered cubic (BCC) structure and grows along the preferred orientation of (110). Therefore, Mo exhibits anisotropic growth properties in the in-plane direction without hexagonal symmetry, which may hinder the growth of highly crystalline AlN [23].

The high crystalline quality of AlN thin films is essential because the crystalline quality is directly related to the important material properties of AlN. For example, it is well known that highly crystalline and highly *c*-axis-oriented AlN thin films are required achieving high piezoelectric coefficients [26]. The best approach to obtain high-quality AlN thin films is epitaxial growth. However, the high temperature required for epitaxial growth may not be compatible with other fabrication conditions, such as those required in complementary metal–oxide–semiconductor (CMOS) processes, and growing AlN thin films epitaxially on the underlying metal electrode layer is practically difficult [27]. We demonstrated the epitaxial growth of highly crystalline AlN thin films on Mo electrode/sapphire substrates at low temperature in our previous study [28]. Highly *c*-axis-oriented epitaxial AlN thin films were successfully prepared even on Mo electrodes, and the possibility of device applications with epitaxial AlN and Mo bottom electrode was suggested.

In this study, we examine the structural characteristics of Mo thin films grown on sapphire (0001) substrates to clarify the reason contributing to the epitaxial growth of AlN thin films on a Mo bottom electrode. First, 100 nm thick Mo thin films deposited on sapphire substrates are prepared for characterizations. The deposition temperature is maintained at 300 °C to ensure the compatibility with CMOS processes. The structural characteristics of the Mo thin films are analyzed via high-resolution X-ray diffraction (HRXRD) 2*θ*–*ω* scans, rocking curves, pole figure measurements, scanning electron microscopy (SEM), and atomic force microscopy (AFM). The Mo thin films on SiO_2_/Si substrates prepared under the same deposition conditions are also characterized for comparison. Consequently, the out-of-plane and in-plane orientation relationships among the AlN thin films, Mo thin films, and sapphire substrates are clearly defined and provided.

## 2. Experimental

To begin, 100 nm thick Mo thin films were deposited by using a pulsed DC magnetron sputtering system. A Mo metal target with a diameter of 100 mm was installed, and a 4-inch sapphire (0001) substrate was placed on the substrate chuck in the deposition chamber. Prior to deposition, the sputtering chamber was evacuated to a base pressure of 5 × 10^−7^ Torr. Subsequently, argon (Ar) gas was injected into the chamber, and the Mo target was sputtered at a pulsed DC power of 300 W. During the deposition, the working pressure and substrate temperature were maintained at 1 mTorr and 300 °C, respectively. The same deposition procedure was performed using a SiO_2_/Si substrate for comparative study. The thickness of the deposited films was confirmed by using a stylus profilometer. The structural properties of the prepared Mo thin films were characterized by HRXRD with CuK_α_ radiation. The preferred out-of-plane orientation and crystalline quality of the Mo thin films were analyzed via 2*θ*–*ω* scans and rocking curve measurements. To identify the in-plane orientations of the films, pole figure measurements were performed. The surface morphology and roughness of the Mo thin films were observed using SEM and AFM, respectively. To demonstrate the epitaxial growth of AlN thin films on Mo electrode/sapphire substrates and define the in-plane orientation relationships among the AlN thin films, Mo thin films, and sapphire substrates, AlN thin films were deposited on a separately prepared Mo thin films/sapphire samples. An Al metal target with a diameter of 100 mm was sputtered in a reactive nitrogen atmosphere at 2.45 mTorr. The power and substrate temperature during the deposition were 500 W and 350 °C, respectively. The epitaxial growth of AlN thin films was confirmed via pole figure measurements.

## 3. Results and Discussion

**Figure 1:**
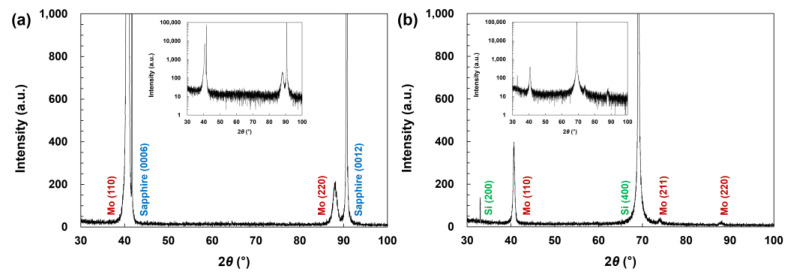
XRD 2*θ–ω* scan pattern of (**a**) Mo thin films grown on sapphire (0001) substrates and (**b**) Mo thin films grown on SiO_2_/Si substrates. Inset in each figure shows the pattern on a logarithmic scale.

The experimental result from the XRD 2*θ*–*ω* scan of the Mo thin films grown on sapphire substrates is shown in Figure 1a. The diffraction intensity on a logarithmic scale is shown in the inset of Figure 1a for better clarity. The diffraction peaks corresponding to Mo (110) and Mo (220) appeared at 2*θ* = 40.7° and 2*θ* = 88.0°, which implies that (110) planes of the Mo crystals were oriented parallel to the substrate surface. The peaks detected at 2*θ* = 41.7° and 2*θ* = 90.7° were attributable to the substrates and corresponded to sapphire (0006) and (0012), respectively. For comparison, the XRD 2*θ*–*ω* scan pattern of the Mo thin films on SiO_2_/Si substrates is shown in Figure 1b. The pattern on a logarithmic scale is also shown in the inset of Figure 1b. The diffraction peaks of Mo (110) and (220) were observed, similar to those of Mo thin films on sapphire substrates. However, the intensities of these peaks were much weaker. These results confirm that (110)-oriented Mo crystals were grown on both substrates, although the crystalline quality was much higher when they were grown on sapphire substrates. In addition, in the case of the Mo thin films on SiO_2_/Si substrates, an additional weak peak from Mo (211) appeared at around 2*θ* = 73.9°, which implies that (211)-oriented Mo crystals coexisted with (110)-oriented crystals in the Mo thin films. The diffraction peaks from the Si substrates corresponding to Si (200) and Si (400) were indicated at 2*θ* = 32.9° and 2*θ* = 69.4°, respectively.

**Figure 2:**
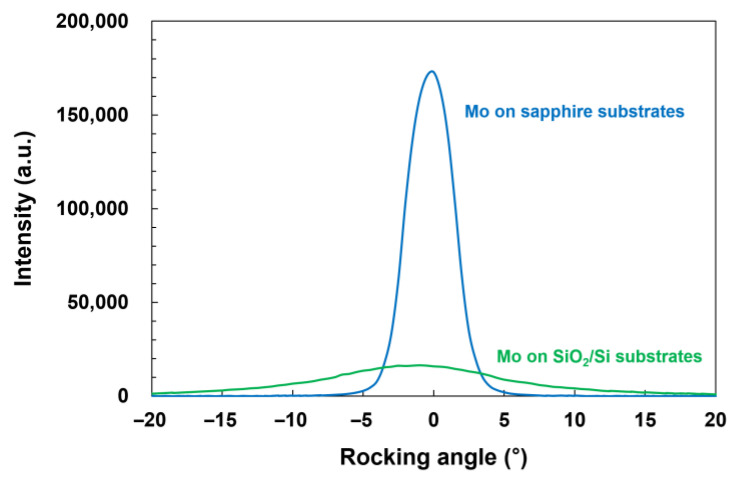
Comparison of XRD rocking curves of Mo (110) reflection from the Mo thin films grown on sapphire (0001) substrates (blue) and from the Mo thin films grown on SiO_2_/Si substrates (green).

Further analyses of the crystalline quality of the (110)-oriented Mo crystals, which were found in both Mo thin films on sapphire and SiO_2_/Si substrates, were performed by XRD rocking curve measurements. The orientation distribution (mosaicity) of the (110)-oriented Mo crystals was obtained directly from the rocking curve. Figure 2 shows the rocking curve of Mo (110) reflection from the Mo thin films on sapphire substrates (blue). The rocking curve of Mo (110) for the Mo thin films on SiO_2_/Si substrates is also shown for comparison (green). The measured rocking curve for the Mo thin films on sapphire substrates showed a significantly stronger and sharper peak than that of the Mo thin films on SiO_2_/Si substrates. The calculated full width at half maximum (FWHM) values of the rocking curves for the Mo thin films on sapphire and SiO_2_/Si substrates were 3.1° and 12.7°, respectively, implying that the orientation degree of the (110)-oriented crystals on sapphire substrates was substantially higher than that of the (110)-oriented crystals on SiO_2_/Si substrates. The weak and broad peak exhibited by the Mo thin films on SiO_2_/Si substrates indicates that the (110)-oriented Mo crystals on SiO_2_/Si substrates were not well oriented and exhibited high mosaicity. These results suggest that the (110)-oriented Mo crystals on sapphire substrates with a higher crystalline quality are better templates for the growth of AlN thin films than the (110)-oriented Mo crystals on SiO_2_/Si substrates.

**Figure 3:**
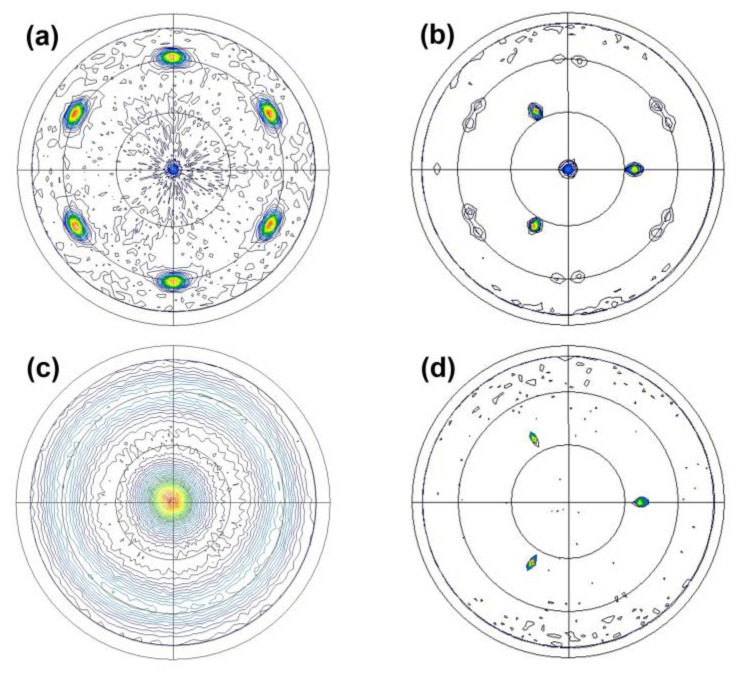
XRD pole figure of (**a**) AlN $\left(10\bar{1}1\right)$ for the AlN thin films grown on Mo/sapphire (0001) substrates, (**b**) Mo (110) for the Mo thin films grown on sapphire (0001) substrates, (**c**) Mo (110) for the Mo thin films grown on SiO_2_/Si substrates, and (**d**) sapphire $\left(10\bar{1}4\right)$.

We previously demonstrated that AlN thin films can be grown epitaxially on Mo electrode/sapphire substrates, which is reconfirmed by the XRD pole figure of AlN $\left(10\bar{1}1\right)$ presented in Figure 3a. The six symmetrical poles separated by an azimuthal angle *φ* of 60° at a tilt angle *ψ* = 61.6° verify that single-domain AlN thin films were grown epitaxially along the *c*-axis even on the Mo thin films. It is assumed that the Mo thin films on sapphire substrates with high crystalline quality served as templates for the epitaxial growth by transferring the crystallographic information of the sapphire substrates to the AlN thin films. To support this assumption, additional analyses of the structural properties of the Mo thin films are required.

Figure 3b shows the pole figure of Mo (110) for the Mo thin films on sapphire substrates. A clear diffraction signal was observed at the center of the pole figure. Because the (110)-oriented Mo crystals were grown on sapphire substrates as confirmed in Figure 1a, the detection of the central pole, which corresponds to the (110)-oriented crystals, was reasonable. The weak twelve poles appearing at a tilt angle *ψ* = 60° indicate other {110} family planes with an interplanar angle of 60°. The in-plane orientations of the (110)-oriented Mo crystals on sapphire substrates were determined from these twelve poles. If the (110)-oriented Mo crystals are aligned in-plane in one direction, then four poles must appear at a tilt angle *ψ* = 60° in the pole figure. Therefore, the twelve poles obtained in our experiments imply that (110)-oriented Mo crystals were grown epitaxially on sapphire substrates, and there existed three in-plane domains rotated with respect to one another by an azimuthal angle *φ* of 120°. In addition, three additional strong poles separated by 120° (*φ*) at around *ψ* = 35.2° appeared. These poles satisfy the angular conditions for (111)-oriented Mo crystals because the interplanar angle between Mo (111) and Mo (110) is 35.26°. Additionally, the number of poles, namely, three, indicates that (111)-oriented single-domain Mo crystals were grown epitaxially on sapphire substrates. Compared with the intensities of these three poles, the intensities of the twelve poles from {110} planes were very weak. Nevertheless, in the XRD 2*θ*–*ω* scan result presented in Figure 1a, the peak corresponding to Mo (111) was not observed, which is attributable to the forbidden X-ray reflection of BCC (111) [29]. In summary, the Mo thin films grown on sapphire substrates exhibited two differently oriented crystals: (110)- and (111)-oriented crystals. The epitaxially grown (110)-oriented crystals comprised three in-plane domains, all of which were detected with extremely weak intensities in the pole figure. By contrast, the (111)-oriented epitaxial crystals were found to be single-domain and considered dominant in the film.

The pole figure of Mo (110) for the Mo thin films on SiO_2_/Si substrates is presented in Figure 3c for comparison. Extremely broad and relatively weak signals were detected close to the center and at around a tilt angle *ψ* = 60°. This indicates that the degree of preferred out-of-plane orientation of the Mo thin films on SiO_2_/Si substrates was not high, although (110)-oriented crystals existed as shown in Figure 1b. Furthermore, the ring pattern at around a tilt angle *ψ* = 60° clearly reveals no preferred in-plane orientation in the Mo thin films, which indicates polycrystalline characteristics. These data confirm again that the Mo thin films on SiO_2_/Si substrates exhibited low crystalline quality and cannot be used as templates for the epitaxial growth of AlN thin films, unlike the Mo thin films on sapphire substrates.

The in-plane orientation relationship between the Mo thin films and sapphire substrates can be identified from the pole figure of sapphire $\left(10\bar{1}4\right)$ presented in Figure 3d. Three sharp poles separated by 120° (*φ*) appeared at around *ψ* = 38.0°. The azimuthal angles *φ* at which the sapphire $\left(10\bar{1}4\right)$ poles appeared were the same as those of the poles corresponding to the (111)-oriented Mo crystals shown in Figure 3b. These results indicate that the $\left[\bar{1}10\right]$ direction of the (111)-oriented Mo crystals was parallel to the $\left[11\bar{2}0\right]$ direction of the sapphire substrates. Similarly, the in-plane orientation between the (110)-oriented Mo crystals and sapphire substrates can be determined. However, unlike the (111)-oriented single-domain Mo crystals, the (110)-oriented Mo crystals comprised three in-plane domains rotated by 120 ° with respect to each other, as mentioned above. By comparing the twelve poles in Figure 3b and the three poles from sapphire $\left(10\bar{1}4\right)$ in Figure 3d, one can verify that the $\left[\bar{1}10\right]$ direction of the (110)-oriented Mo crystals was parallel to the $\left[11\bar{2}0\right]$, $\left[1\bar{2}10\right]$, and $\left[\bar{2}110\right]$ directions of the sapphire substrates. The in-plane orientation relationship between the epitaxial AlN thin films and sapphire substrates can be similarly identified by comparing the pole figure of AlN $\left(10\bar{1}1\right)$ in Figure 3a with that of sapphire $\left(10\bar{1}4\right)$ in Figure 3d. A 30° shift in the azimuthal angle *φ* was found between the six symmetrical poles of AlN $\left(10\bar{1}1\right)$ and the three symmetrical poles of sapphire $\left(10\bar{1}4\right)$. Therefore, the in-plane orientation relationship between the AlN thin films and sapphire substrates can be defined as AlN $\left[10\bar{1}0\right]$//sapphire $\left[11\bar{2}0\right]$. The out-of-plane and in-plane orientation relationships among the AlN thin films, Mo thin films, and sapphire substrates can be summarized as shown in Table 1.

**Figure 4:**
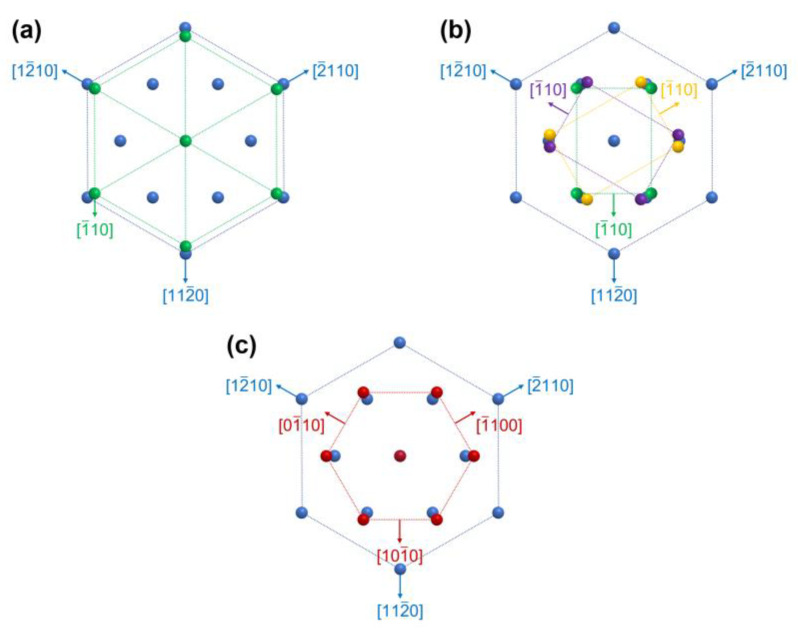
In-plane orientation relationship between (**a**) (111)-oriented Mo and sapphire, (**b**) (110)-oriented Mo and sapphire, and (**c**) (0001)-oriented AlN and sapphire.

Figure 4a,b illustrate the in-plane orientation relationships between the Mo thin films and sapphire (0001) substrates; (111)-oriented Mo crystals in Figure 4a and (110)-oriented Mo crystals in Figure 4b. As mentioned above, Mo thin films typically grow along the preferred orientation of (110). However, on sapphire (0001) substrates with the hexagonal symmetry, it can be preferentially grown along (111) plane because of the better atomic fitting; therefore, the (111)-oriented epitaxial crystals were dominant in the Mo thin films deposited on sapphire substrates. Figure 4c shows the in-plane orientation relationship between AlN thin films and sapphire (0001) substrates. It is well known that the epitaxial growth of AlN thin films can be achieved on sapphire (0001) substrates with an in-plane rotation of 30° because this rotation can decrease the lattice mismatch from approximately 35% to 13% [30,31]. The AlN thin films grown on Mo/sapphire (0001) substrates in our experiments showed the same in-plane rotation, which implies that the crystallographic information of the sapphire substrates was transferred to the AlN even in the presence of interlayered Mo thin films. These results suggest that the highly ordered Mo thin films formed on sapphire substrates can be used as templates for the epitaxial growth of AlN thin films and that they do not prevent the transferring of the crystallographic information on the sapphire substrates.

**Figure 5:**
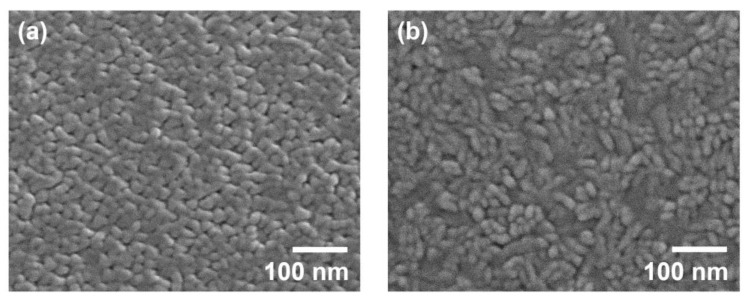
SEM images of the surface morphology of the Mo thin films grown on (**a**) sapphire (0001) substrates and (**b**) SiO_2_/Si substrates.

Figure 5a shows the surface morphology of the Mo thin films grown on sapphire substrates observed by SEM. The surface was uniform and comprised nanocrystalline grains with an average size ranging from 20 to 30 nm. Numerous triangular facets corresponding to Mo (111) planes were clearly observed. This result is consistent with the findings from the pole figure analysis shown in Figure 3b, i.e., epitaxially grown (111)-oriented crystals existed in the Mo thin films. However, as shown in Figure 5b, the surface morphology of the Mo thin films on SiO_2_/Si substrates examined using SEM was completely different. The surface morphology was not homogeneous, and disordered irregular grains with elongated shapes were observed. As mentioned above, the Mo thin films on SiO_2_/Si substrates exhibited low crystalline quality and a random in-plane orientation, which is similarly confirmed again in Figure 5b.

**Figure 6:**
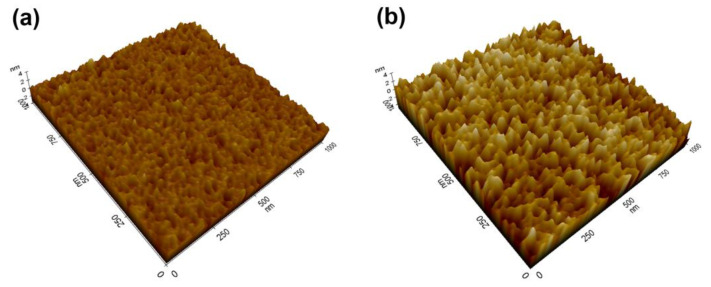
AFM images of the Mo thin films grown on (**a**) sapphire (0001) substrates and (**b**) SiO_2_/Si substrates.

Figure 6a,b show the AFM images (1 µm × 1 µm) of the Mo thin films on sapphire and SiO_2_/Si substrates, respectively. The result reconfirmed that the surface morphology of the Mo thin films on sapphire substrates was uniform and smooth. The root mean square (RMS) roughness of the surface was measured to be 0.46 nm. However, as expected, the surface of the Mo thin films on SiO_2_/Si substrates was relatively non-uniform, and the RMS roughness was 1.1 nm, which was more than double that of the Mo thin films on sapphire substrates. These results are consistent with the findings from the SEM analysis and support the conclusion that the Mo thin films grown on sapphire substrates can serve as templates for the epitaxial growth of AlN thin films.

## 4. Conclusions

The growth of high-quality AlN thin films on Mo electrodes is required in many cases. We successfully demonstrated the epitaxial growth of highly crystalline and highly *c*-axis-oriented AlN thin films on Mo electrode/sapphire (0001) substrates. To investigate the reason contributing to the epitaxial growth of AlN thin films on Mo electrodes, the structural analysis of the Mo thin films grown on sapphire (0001) substrates was performed. The structural characteristics of the Mo thin films such as the crystalline quality, out-of-plane orientation, and in-plane orientation were analyzed via XRD 2*θ*–*ω* scans, rocking curves, and pole figure measurements. The surface morphology and roughness of the Mo thin films were observed using SEM and AFM. It was confirmed that the Mo thin films grown on sapphire substrates comprised two differently oriented crystals; the dominant (111)-oriented single-domain crystals and the recessive (110)-oriented crystals with three in-plane domains rotated by 120° with respect to each other. These results differed significantly from those obtained for the Mo thin films on SiO_2_/Si substrates prepared for a comparative study. The Mo thin films on SiO_2_/Si substrates exhibited low crystalline quality and polycrystalline characteristics. The defined out-of-plane and in-plane orientation relationships among the AlN thin films, Mo thin films, and sapphire substrates can be expressed as follows:

$$\begin{array}{r} \mathrm{AlN}\;(0001)\;//\;\mathrm{Mo}\;(111)\;//\;\mathrm{Sapphire}\;(0001)\;\mathrm{with}\;\mathrm{AlN}\;[10\bar{1}0]\;//\;\mathrm{Mo}\;[\bar{1}10]\;//\;\mathrm{sapphire}\;[11\bar{2}0],\\ \mathrm{AlN}\;(0001)\;//\;\mathrm{Mo}\;(110)\;//\;\mathrm{Sapphire}\;(0001)\;\mathrm{with}\;\mathrm{AlN}\;[10\bar{1}0]\;//\;\mathrm{Mo}\;[\bar{1}10]\;//\;\mathrm{sapphire}\;[11\bar{2}0],\\ \mathrm{AlN}\;[0\bar{1}10]\;//\;\mathrm{Mo}\;[\bar{1}10]\;//\;\mathrm{sapphire}\;[1\bar{2}10],\\ \mathrm{AlN}\;[\bar{1}100]\;//\;\mathrm{Mo}\;[\bar{1}10]\;//\;\mathrm{sapphire}\;[\bar{2}110]. \end{array}$$

Based on the out-of-plane and in-plane orientation relationships among the AlN thin films, Mo thin films, and sapphire substrates, one can conclude that highly ordered Mo thin films on sapphire substrates can be used as templates for the epitaxial growth of AlN thin films by transferring the crystallographic information of the sapphire to the AlN. This study is expected to contribute to the development of MEMS devices that require highly crystalline AlN thin films/Mo electrode structures, and we will further investigate the device applications in our future work. Studying the influence of the thickness of Mo films on the epitaxial growth of AlN films is also suggested for future research. 

## Figures and Tables

**Table 1:** Out-of-plane and in-plane orientation relationships among AlN thin films, Mo thin films, and sapphire substrates.

Out-of-Plane Relationship	In-Plane Relationship
AlN (0001)//Mo (111)//Sapphire (0001)	$\mathrm{AlN}\;\left[10\bar{1}0\right]$ $//\mathrm{Mo}\;\left[\bar{1}10\right]$ $//\mathrm{sapphire}\;\left[11\bar{2}0\right]$
AlN (0001)//Mo (110)//Sapphire (0001)	$\mathrm{AlN}\;\left[10\bar{1}0\right]$ $//\mathrm{Mo}\;\left[\bar{1}10\right]$ $//\mathrm{sapphire}\;\left[11\bar{2}0\right]$
	$\mathrm{AlN}\;\left[0\bar{1}10\right]$ $//\mathrm{Mo}\;\left[\bar{1}10\right]$ $//\mathrm{sapphire}\;\left[1\bar{2}10\right]$
	$\mathrm{AlN}\;\left[\bar{1}100\right]$ $//\mathrm{Mo}\;\left[\bar{1}10\right]$ $//\mathrm{sapphire}\;\left[\bar{2}110\right]$

## Data Availability

Not applicable.
